# The Impact of Tillage and Crop Residue Incorporation Systems on Agrophysical Soil Properties

**DOI:** 10.3390/plants12193386

**Published:** 2023-09-25

**Authors:** Vaida Steponavičienė, Aušra Rudinskienė, Giedrius Žiūraitis, Vaclovas Bogužas

**Affiliations:** Department of Agroecosystems and Soil Sciences, Vytautas Magnus University, K. Donelaičio Street 58, 44248 Kaunas, Lithuania

**Keywords:** long-term experiment, shear strength, soil aggregate stability, straw return, cover crop

## Abstract

A long-term field experiment has been ongoing since 1999 at the Experimental Station of Vytautas Magnus University’s Agriculture Academy. According to the latest edition of the International Soil Classification System, the soil in the experimental field can be classified as Planosol, with a silty medium-loam texture at a depth of 0–20 cm and a silty light-loam texture at a depth of 20–40 cm. Studies were carried out on winter wheat crops in 2014, 2017, and 2023. This research aimed to assess how different long-term tillage systems impact soil shear strength and aggregate stability, their interconnection, and the effect of crop residues on soil stability. The treatments were arranged using a split-plot design. In a two-factor field experiment, straw was removed from one part of the experimental field, while the entire straw yield was chopped and spread at harvest in the other part (Factor A). The subplot factor (Factor B) included three different tillage systems: conventional deep ploughing, cover cropping for green manure with no tillage, and no tillage. The soil samples were analyzed at the Laboratory of Agrobiology at Vytautas Magnus University’s Agriculture Academy. The findings indicated that the long-term application of reduced tillage significantly increased the soil shear strength. Shallower tillage depths led to a higher soil shear strength, while the effect of spreading plant residues was relatively lower. The long-term tillage of different intensities, spreading plant residues, and catch crop cultivation for green manure did not significantly affect the soil structure. However, the soil structural stability was found to be highly dependent on soil tillage. Cover cropping for green manure with no tillage and no tillage alone positively affected the soil aggregate stability in the upper 0–10 cm and 10–25 cm layers. The correlation–regression analysis showed that, in the top 0–10 cm and 10–25 soil layers, there were moderate to strong correlations between the soil structural stability, soil shear strength, and the effect of crop residues on soil stability.

## 1. Introduction

Preserving and enhancing soil functions are essential aspects of sustainable agricultural practices. The implementation of long-term crop rotation and tillage practices significantly influences the soil environment by introducing inputs and disturbances that impact the soil quality. The long-term impact of tillage on agroecosystems has been widely acknowledged. Conservation tillage systems that minimize soil disturbance, such as reduced or no tillage and residue retention or mulching, are often advocated to improve agricultural sustainability. Previous studies have demonstrated the positive impact of reduced tillage on soil properties [1]. Conservation agriculture encompasses a set of principles aimed at implementing and developing sustainable technologies that ensure sufficient plant residues on the soil surface for erosion control, reduced water evaporation and surface runoff, optimized rainfall utilization, and the long-term enhancement of physical, chemical, and biological soil properties, leading to stable and sustained yields [2].

The importance of soil health has gained exponential awareness and interest over the past decade, with excessive tillage identified as a significant contributor to soil degradation, particularly erosion [3]. The soil physical properties of a long-term no-till system undergo drastic changes with subsequent tillage, rendering previously valid soil quality indicators invalid [4]. Conservation tillage systems have been shown to have a positive impact on soil porosity and aggregate stability across various soil types and climates [5]. However, the benefits of conservation tillage practices, including reduced soil disturbance, cover cropping, and residue retention, appear to be site-specific and influenced by the post-adoption period, climate conditions, properties of the previous management system, and the choice of cover crop species [6].

Regarding soil structure, it has been observed that different tillage technologies do not significantly alter the soil aggregate structure. The most substantial increases in soil aggregate stability at sizes above 1 mm (average increases of 74.3% and 96.3%) and above 0.25 mm (average increases of 14.6% and 20.3%) have been observed in shallowly loosened or untilled soil (direct drilling) compared with conventionally ploughed soil at the beginning of the vegetation period. Similar trends have been observed at the end of the vegetation period, although the impact of tillage diminished [7].

Soil hardness is another important determinant of soil quality. Smaller soil aggregates result in higher soil hardness. The soil hardness increases as the soil density rises and the moisture content decreases [8]. Direct drilling is associated with higher soil hardness compared with deep and shallow ploughing. Immediately after sowing, the topsoil (0–10 cm) exhibits similar hardness in both shallow ploughing and deep ploughing treatments, while directly drilled soil shows 49–54% higher hardness [9].

Sustainable agricultural practices play a pivotal role in upholding and enhancing soil functions. The enduring application of techniques such as crop rotation and tillage carries the potential to shape the soil environment by introducing inputs and disturbances that intricately affect the soil quality. The ramifications of tillage reverberate through agroecosystems in the long term [10,11].

The excessive compaction of untilled topsoil, which leads to a decrease in the physical quality of soil, is considered a primary cause of yield reductions [12]. This issue is particularly problematic for weakly structured soils in humid temperate climates [13,14].

These recent findings further emphasize the importance of implementing sustainable agricultural practices, such as conservation tillage systems, to enhance soil quality, improve crop productivity, and ensure the long-term sustainability of agricultural systems [15]. Conservation agriculture practices have emerged as pivotal agents that shape not only the structural robustness of soil aggregates, but also the intricate interactions of nutrient dynamics and agricultural sustainability. As conservation agriculture transcends geographical boundaries, its potential to influence soil health, nutrient availability, and overall agricultural productivity becomes increasingly evident. The nexus between these practices and the intricate stability of soil aggregates underscores the imperative of adopting comprehensive approaches for land management that account for a range of interconnected factors [16,17,18].

Soil shear strength and aggregate stability are closely related because they both involve interactions between soil particles and the forces acting upon them. The stability of soil aggregates influences how soil responds to shear forces, and, conversely, how the shear forces experienced by soil can affect the stability of its aggregates [19].

Soil aggregates are formed by the binding of individual soil particles through various mechanisms, including organic matter, clay minerals, and microbial activity. These aggregates create a network of interconnected pores, enhancing the soil’s structural integrity. When shear forces are applied to the soil, aggregates play a crucial role in resisting the movement and displacement of particles. Well-formed aggregates offer greater resistance to shear stress compared with individual loose particles, as the internal cohesion of the aggregates helps them maintain their structure [20]. Plant residues contribute to soil organic matter, which, in turn, affects the soil structure and stability. Soil organic matter derived from plant residues can act as a binding agent, promoting the formation and stability of soil aggregates [21,22]. The contribution of plant residues and the subsequent impact on soil structure and stability is another facet warranting attention. As our research delineates, plant residues undergo decomposition to yield organic matter, which acts as a cohesive agent, cementing soil particles into aggregates. This transformative process was extensively researched into the decomposition dynamics of varied plant residues and their respective influence on soil aggregation. They discerned that high lignin-containing residues, due to their slow decomposition, induced a more lasting impact on soil aggregation than residues with rapid decomposition rates [23,24].

Soil shear strength is influenced by soil texture, moisture content, and soil structure. Plant residues can indirectly impact shear strength by affecting soil compaction and aggregate stability. Soils with stable aggregates tend to have a higher shear strength.

This research aims to assess how different long-term tillage systems impact the soil shear strength and aggregate stability, their interconnection, and the effect of crop residues on soil stability. Tillage practices can vary from conventional ploughing, where the soil is deeply turned over, to reduced tillage or no-tillage systems, where soil disturbance is minimized or eliminated. Each tillage system can lead to a distinct impact on the soil structure, organic matter distribution, and soil compaction, ultimately influencing the soil shear strength.

## 2. Results and Discussion

### 2.1. Soil Shear Strength

Shear strength describes the amount of energy needed to shear soil. It also refers to the energy needed to pull an implement such as a planter through the soil. Shear strength is often determined in the field or on undisturbed soil samples. We found three studies that measured shear strength under different tillage regimes [25]. When direct drilling was implemented, it induced changes in closely related soil characteristics, wherein alterations in one characteristic led to corresponding changes in others [26].

**Figure 1:**
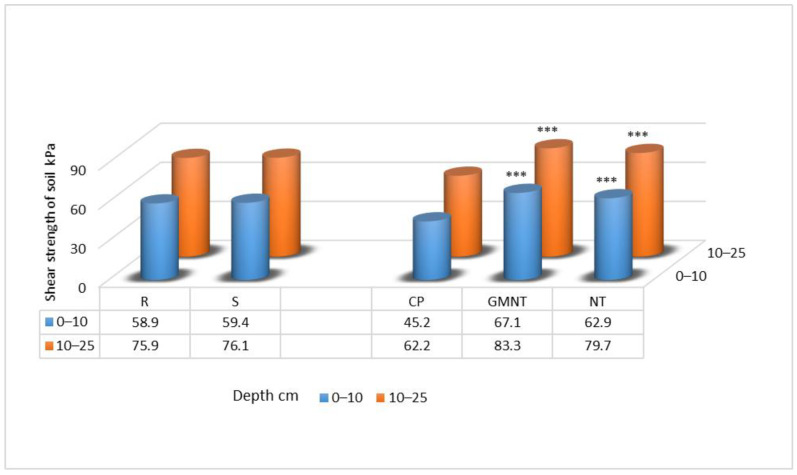
Shear strength of the soil in spring 2014. Notes: Significant differences at *** *p* ≤ 0.001; Fisher’s LSD test vs. control. Factor A: R—straw removed (control); S—straw chopped and spread. Factor B: CP—conventional deep ploughing (control); GMNT—cover cropping for green manure with no tillage; NT—no tillage, direct drilling.

In 2014, at the beginning of winter wheat vegetation, the effect of tillage systems on the soil shear strength was significant both in the upper (0–10 cm) and lower (10–25 cm) layers (Figure 1). Compared with conventional ploughing, the rest of the no-till systems significantly increased the soil shear strength.

In line with these findings, recent research has shed further light on the diverse impact of various tillage methods on soil strength and structure. Their study compared different tillage practices with conventional ploughing and revealed that all other tillage systems significantly increased the soil shear strength [27].

Moreover, a comprehensive study focusing on the impact of tillage systems on soil shear strength demonstrated results that are in line with the findings of [28], showing that all alternative tillage methods led to a significant increase in the soil shear strength compared with conventional ploughing.

Furthermore, a meta-analysis provided additional evidence supporting the effectiveness of alternative tillage practices in enhancing both the soil shear strength and sustainability. The meta-analysis synthesized data from multiple studies and reinforced the notion that adopting innovative tillage techniques can positively influence soil properties and contribute to improved agricultural practices [29].

Overall, the growing body of research emphasizes the importance of considering different tillage strategies and their impact on the soil shear strength and structure. These findings play a crucial role in promoting sustainable agricultural practices and soil management strategies, ensuring long-term soil health and productivity. As researchers continue to explore the complexities of soil–tillage interactions, farmers and land managers can make informed decisions to preserve and improve soil quality, contributing to a more resilient and sustainable agricultural system.

**Figure 2:**
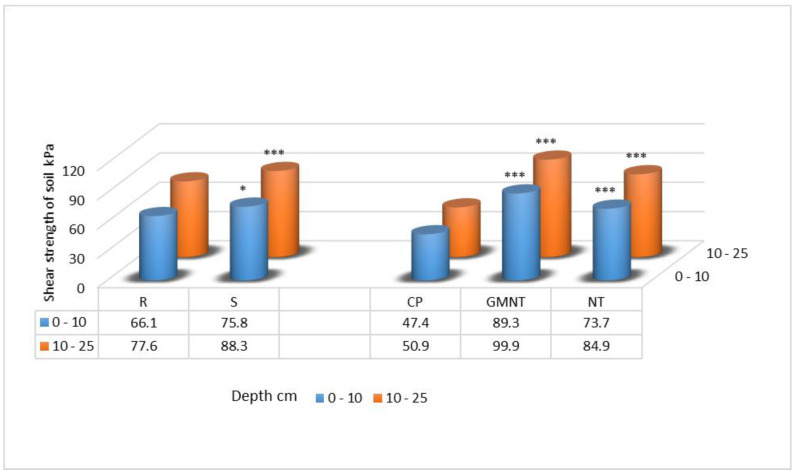
Shear strength of the soil in spring 2017. Notes: Significant differences at * 0.010 < *p* ≤ 0.050; *** *p* ≤ 0.001; Fisher’s LSD test vs. control. Other explanations are as per Figure 1.

The results show that the soil shear strength in the upper (0–10 cm) soil layer was significantly (11.6%) higher when straw was present compared with when it was not. Similarly, in the deeper soil layer (10–25 cm), the shear strength was 11.1% higher in the presence of straw (Figure 2). These findings highlight the beneficial impact of incorporating straw into soil on the shear strength [30].

Furthermore, we also investigated the effect of different tillage systems on the soil shear strength. It was observed that tillage systems had a significant influence in both the 0–10 cm and 10–25 cm soil layers. In the upper (0–10 cm) layer, the shear strength increased from 14.6% to 64.5% across different tillage systems, while in the deeper (10–25 cm) layer, the increase ranged from 49.8% to 71.7%.

These findings align with other recent studies emphasizing the positive impact of straw incorporation and alternative tillage practices on the overall soil health [31,32].

**Figure 3:**
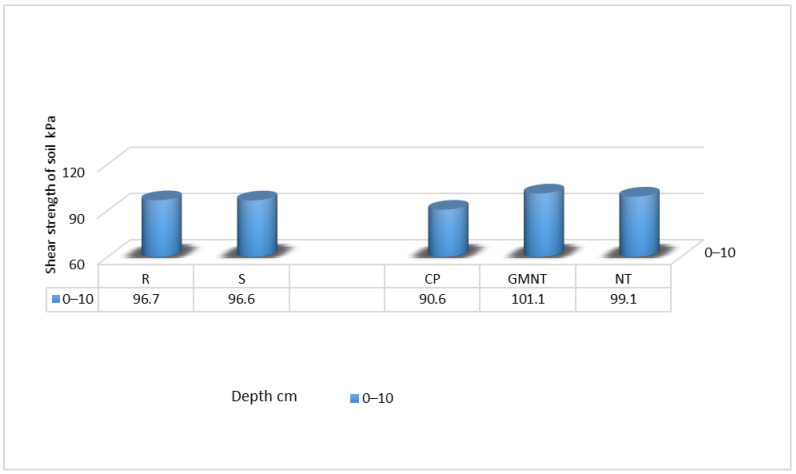
Shear strength of the soil in spring 2023. Notes: No significant differences at *p* > 0.05; Fisher’s LSD test vs. control. Other explanations are as per Figure 1.

In 2023, this study faced challenges in measuring the soil shear strength beyond the upper (0–10 cm) layer due to lower-than-usual precipitation in May, resulting in excessively hard soil conditions (Figure 3). The scarcity of moisture in the soil made it difficult to conduct further measurements; thus, only the upper layer was evaluated.

Despite the limited measurements, this study yields notable insights. It was found that different tillage practices had a considerable impact on the soil shear strength in the upper (0–10 cm) layer. Among the tillage methods, the direct drilling approach exhibited an 11.3% increase in the shear strength compared with conventional ploughing, suggesting that this method could be particularly advantageous in drought-prone or low-moisture conditions.

These findings show the importance of considering moisture levels and selecting the right tillage practices to effectively manage the soil shear strength. This study highlights that the soil moisture content can significantly influence the soil shear strength, and, in dry conditions, certain tillage methods may be more suitable to maintain soil integrity.

The research further reinforces the impact of tillage systems on various soil properties. These studies emphasize the need for context-specific approaches in soil management, taking into account regional climate patterns, soil types, and crop requirements. By tailoring tillage practices to local conditions, farmers and land managers can optimize soil health and productivity, contributing to sustainable and resilient agricultural systems [33].

In conclusion, the 2023 study’s findings shed light on the significance of moisture levels in influencing the soil shear strength, with direct drilling showing promise under drought conditions. Incorporating knowledge from previous research further emphasizes the importance of adopting tailored tillage practices to improve soil properties and support sustainable agricultural practices [34].

Taken together, the three years of experimental data show a significant increase in the soil shear strength over time with reduced tillage. It should be noted that the lower the tillage depth, the greater the increase in the soil shear strength.

The influence of straw spreading on soil penetration and shear strength was assessed, specifically in drier and hotter years. It is under these climatic conditions that the impacts of straw spreading on soil penetration and shear strength were examined, revealing valuable insights into its impact on soil properties. These findings are in line with prior research also highlighting the beneficial impact of reduced tillage practices on soil strength and the importance of considering specific climatic conditions when managing agricultural systems [35,36]. The shear strength did not differ among NT, moldboard plow, chisel plow, or disk systems in the three studies, further suggesting that NT does not necessarily cause soil consolidation and increase soil strength. In contrast, by reducing the surface residue cover, CT may cause the formation of soil crusts, which can increase soil strength [37]. The crusting or cracking of soils can be a problem in agricultural fields. Raindrops striking the bare soil surface can break down soil aggregates and induce the formation of surface seals. Upon rapid drying and consolidation, surface seals form crusts [38]. Crusts have a higher shear strength and penetration resistance than the soil layers below. No-till practices reduce the formation of crusts by maintaining the surface residue cover. Residue mulch intercepts erosive raindrops, slows soil drying, and reduces surface sealing and crusting. Similar to crusting, the use of NT can reduce the formation of cracks through the retention of crop residues and reduced evaporation. In [39], it was found that cracks under NT had a smaller surface area and volume than CT in a Vertisol. The same study found that the depth of soil cracks decreased as the amount of water in the soil increased. When soils dry quickly, large cracks may form, particularly in clayey soils with high shrink–swell potential (i.e., claypan soils and Vertisols). While cracks can be pathways for water recharge, they can reduce soil–plant root contact and increase nutrient loss to deeper layers. Soil consistency (Atterberg limits), which refers to soils’ resistance to deformation and compaction, can also be affected by tillage systems, although related studies are very scarce. In a long-term experiment (>20 years) in a silt loam in Ohio, NT exhibited a greater soil water content at liquid and plastic limits compared with moldboard plow [31]. Similarly, across four soils in the central Great Plains, NT soils retained more water at the liquid limit than CT [40]. The greater consistency limits with NT are attributed to an increased soil organic C concentration, which can provide elasticity and resistance to deformation [41]. The increased consistency with NT indicates that these soils can be trafficked when wetter than CT or RT without inducing significant compaction. It further indicates that NT soils can be more resilient to traffic-induced compaction than conventionally tilled soils. Soil consistency can thus be related to Proctor and compression index tests.

### 2.2. Soil Aggregate Stability

Soil aggregate stability is a vital factor that influences crop yield, especially when coupled with good tillage practices [42]. The conducted research showed that conventional tillage practices reduced the macroaggregate fraction at a depth of 0–20 cm. This reduction in the >0.25 mm aggregate fraction is attributed to the mechanical disruption of the soil structure macroaggregates caused by frequent tillage operations, resulting in reduced aggregate stability.

The type of soil plays a significant role in determining the best tillage practices. Reduced tillage methods are particularly beneficial because they can help to minimize moisture loss and maintain optimal soil temperature, which is crucial for creating an ideal seedbed. However, the soil structure can be degraded due to various factors, including mechanical, physical, biological, and chemical elements. Cultivation, driving over the soil, and heavy precipitation can mechanically dismantle the soil structure, while the leaching of calcium from the upper soil layers and decreases in the humus content can contribute to soil degradation. As a result, soil-stable aggregates lose their adhesive properties and begin to decompose. To combat soil degradation, it is crucial to control the factors leading to soil structure deterioration and make efforts to enrich the soil with organic matter [43,44].

**Figure 4:**
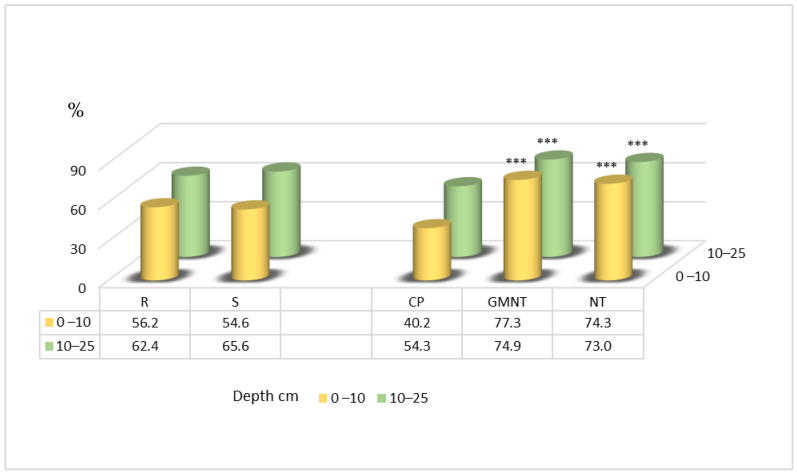
Soil aggregate stability in spring 2014. Notes: Significant differences at *** *p* ≤ 0.001; Fisher’s LSD test vs. control. Other explanations are as per Figure 1.

The results of this research, which has been carried out since 2014, show that the soil aggregate stability in the 0–10 cm soil layer was significantly higher with measures such as direct drilling compared with conventional ploughing. The increase ranged from 1.8-fold to 2-fold (Figure 4). In the 10–25 cm soil layer, a similar significant increase in the soil aggregate stability was observed, with the treatments resulting in 1.4-fold greater stability compared with conventional ploughing. Notably, straw spreading did not have a significant influence on the soil aggregate stability in this study.

These findings are in line with other research also highlighting the importance of good tillage practices and their impact on the soil aggregate stability [45].

**Figure 5:**
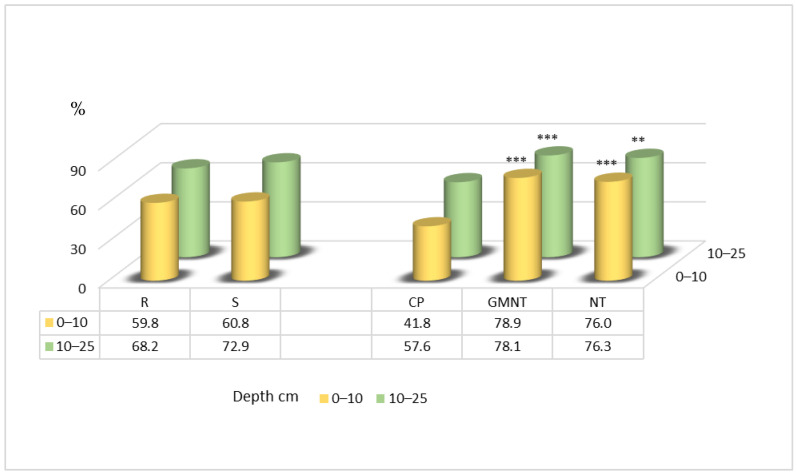
Soil aggregate stability in spring 2017. Notes: Significant differences at ** 0.001< *p* ≤ 0.010; *** *p* ≤ 0.001; Fisher’s LSD test vs. control. Other explanations are as per Figure 1.

The data from 2017 showed significantly higher soil aggregate stability in the 0–10 cm layer (Figure 5) with the direct drilling of plant residues and direct drilling techniques compared with conventional ploughing, by 37 and 34 percentage points, respectively.

These findings highlight the dynamic nature of the soil’s response to different tillage practices and reinforce the notion that good tillage methods can significantly influence soil properties. The substantial improvements in the soil aggregate stability observed with the specified treatments underscore their potential to enhance the soil structure, reduce erosion risks, and improve water infiltration, similar to the results obtained by other authors [46].

This study emphasizes the importance of considering year-to-year variations in the impact of tillage practices on soil aggregate stability. Soil health and stability are influenced by various factors, including weather conditions, cropping patterns, and management practices, making it essential to evaluate data across multiple years to gain a comprehensive understanding of soil responses.

In summary, the 2017 data reinforce the positive impact of specific tillage practices, such as the direct drilling of plant residues and direct drilling, on the soil aggregate stability in the 0–10 cm soil layer. These findings further support the significance of adopting sustainable tillage strategies to maintain and improve soil health, providing valuable insights for informed decision making in agricultural land management.

**Figure 6:**
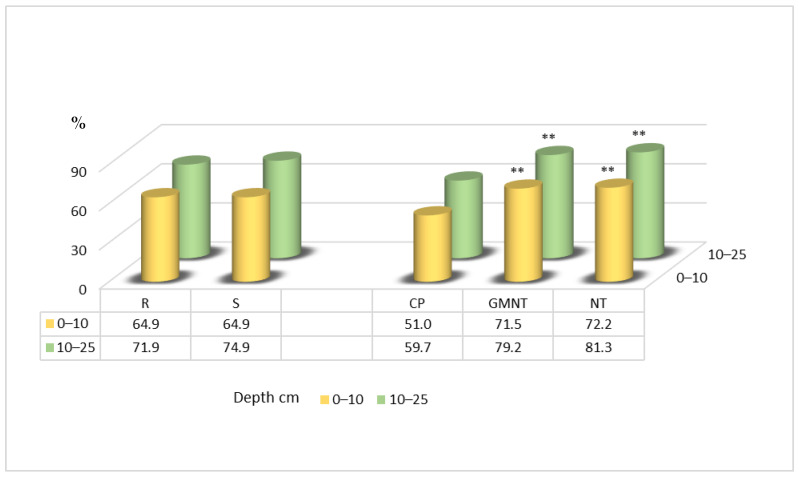
Soil aggregate stability in spring 2023. Notes: Significant differences at ** 0.001< *p* ≤ 0.010; Fisher’s LSD test vs. control. Other explanations are as per Figure 1.

According to the results of the 2023 research, significant increases in the soil aggregate stability were observed in both layers of the soil (Figure 6) with two specific treatments. The treatment involving the direct drilling of plant residues showed 20 and 20 percentage point increases in stability, while direct drilling alone resulted in 21 and 22 percentage point increases in stability, both compared with conventional ploughing.

Moreover, this study reveals that the soil aggregate stability was the lowest when the soil was ploughed deeply or shallowly each year. However, the incorporation of straw residues tended to increase the aggregate stability. These trends are in line with similar research highlighting the consistent impact of different tillage practices on the soil aggregate stability [47,48].

Additionally, the positive effect of direct drilling on the soil structure was evident, as it significantly reduced the number of small aggregates and increased the number of stable aggregates not only in the upper (0–10 cm) soil layer, but also in the lower (10–25 cm) soil layer. This finding indicates that the increase in large aggregates in no-till soils is associated with the presence of a high level of crop residues on the soil surface and minimal soil disturbance, promoting soil structural stability [49]. No-till practices could affect the structural quality of soil differently from CT through reduced soil disturbance and the maintenance of a permanent residue cover. Improved soil structural quality is essential to many soil processes and properties. For example, it can affect soil erosion (i.e., soil detachment), surface sealing and crusting, pore size distribution, water infiltration and retention, compaction risk, and the protection of organic matter and nutrients. The immediate effects of diverse tillage methods and plant residue retention on soil physical characteristics and greenhouse gas emissions were investigated. Their inquiry unveiled the dynamic interplay that underpins soil management strategies and the stability of soil aggregates. It became evident that the manipulation of these practices not only orchestrates shifts in the soil structure, but also resonates through the intricate process of aggregate formation and stability. These dynamics are poised to yield far-reaching consequences, potentially impacting greenhouse gas emissions, which is a facet of paramount environmental concern [50,51].

Significant carved niches were assessed by exploring the enduring implications of prolonged tillage practices on soil aggregates and the carbon they host. The revelations underscore the intricate correlation between the duration of tillage practices and the physical resilience of soil aggregates. This vantage point provides valuable insight into how protracted tillage practices possess the potential to remodel the soil structure, consequently influencing the stability of the aggregates and their prowess in sequestering carbon—a critical facet of soil’s role in mitigating carbon dioxide in the atmosphere [52].

The meticulous evaluation of methods used to quantify soil aggregate stability brings to the fore the pivotal role played by precise measurement techniques. This work accentuates the paramount significance of accuracy in unraveling the nuances of soil aggregate stability. This nuanced approach is pivotal in deciphering the influence of a gamut of factors, spanning from tillage techniques to the incorporation of organic matter, on the inherent structural soundness of soil aggregates [53].

Soil shear strength and aggregate stability are closely related because the forces involved in shear stress can affect the integrity of soil aggregates, and the stability of aggregates can influence how well the soil can resist shear forces. Maintaining soil aggregates through practices that promote good soil structure and organic matter content can contribute to both improved shear resistance and enhanced aggregate stability [19,54]. The correlation–regression analysis showed moderate to strong correlations in the top (0–10 cm) layer of soil between the soil structural stability and shear resistance in 2014 (*r* = 0.95; *p* ≤ 0.01), 2017 (*r* = 0.93; *p* ≤ 0.05), and 2023 (*r* = 0.95; *p* ≤ 0.01), as well as in the lower soil layer (10–25 cm) in 2014 (*r* = 0.88; *p* ≤ 0.01) and 2017 (*r* = 0.83; *p* ≤ 0.05).

### 2.3. Content of Plant Residues on the Soil Surface and in the Soil

Plant residues on the soil surface and within the soil play crucial roles in shaping soil health and fertility. The decomposition and incorporation of these residues into the soil influence various soil properties and processes.

The content of plant residues on the soil surface and within the soil profile plays a pivotal role in determining various soil attributes and functionalities. These residues, often comprising crop residues, fallen leaves, stems, and root systems, impact a range of soil characteristics, from its physical structure to its chemical properties [55]. The incorporation or burial of plant residues leads to a higher level of microorganism activity compared to when they are left on the surface, as the respiration rate is 60% higher. The way in which crop residues are incorporated is an important factor in the decomposition of organic matter and nutrient dynamics in the soil, as well as in the overall structure and durability of the soil. Deeply buried organic residues are forced to decompose under anaerobic conditions, which can lead to the formation of phytotoxic compounds. Leaving plant residues on the soil surface reduces evaporation; as such, more water remains in the soil [56].

Plant residues, such as crop residues and root materials, are sources of organic matter. When these residues decompose, they release essential nutrients into the soil, which also impact the soil’s agrophysical properties [57].

The data obtained in 2014 (Table 1) show that the incorporation of straw increased the content of plant residues on the soil surface by 2-fold compared with the soil without straw. A comparison of different tillage systems with deep ploughing revealed significant differences: in the direct drilling of plant residues and direct drilling techniques, the contents of plant residues were 4.4-fold and 5.7-fold higher than when the deep ploughing treatment was used, respectively. The data obtained in 2017 exhibit very similar results, indicating that straw incorporation increased the content of plant residues on the soil surface by 1.7-fold. In the direct drilling of plant residues and direct drilling techniques, the contents of plant residues were 10.7-fold and 13.6-fold higher, respectively.

The data from other researchers are highly correlated with ours. The incorporation of plant residues into the soil helps to improve the soil structure by promoting the formation of aggregates. These aggregates reduce erosion and create a favorable environment for root growth. It has been demonstrated that organic matter from plant residues can enhance soil aggregation and stability [58].

**Figure 7:**
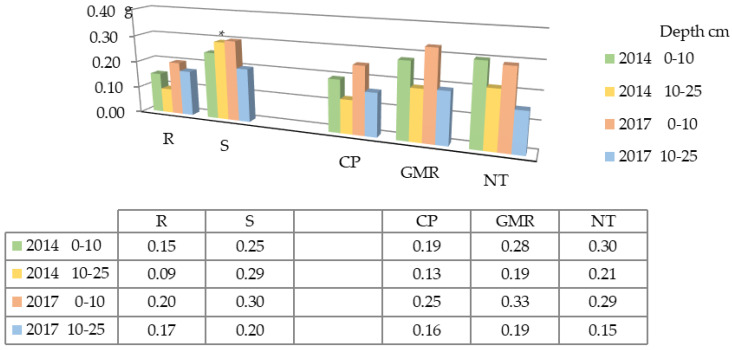
Content of plant residues in the soil (100 g sample) in 2014 and 2015. Notes: Significant differences at * *p* ≤ 0.05 > 0.01; Fisher’s LSD test vs. control. Other explanations are as per Figure 1.

When assessing the content of plant residues in the soil in 2014 (Figure 7), straw incorporation did not have significant effect on the upper 0–10 cm soil layer; however, a significant increase was determined in the bottom 10–25 cm soil layer, where the content of plant residues was 2.8-fold higher than in the soil without straw.

In the experimental year of 2017, straw spreading did not have a significant effect on the upper 0–10 cm soil layer; however, significant influences were observed in the 10–25 cm soil layer. In summary, it can be concluded that when straw is spread, significantly more plant residues remain in the soil when they are incorporated at a 10–25 cm depth, where they are more slowly mineralized. In the direct drilling of plant residues and direct drilling techniques, no significant differences were found in 2014 nor in 2017; only differences in trends were found. Similar to our emphasis on plant residues as key players in soil health, one study expounded on the potential of straw and other crop residues in enhancing the soil aggregate stability. Other researchers have underlined the importance of residues not just as sources of nutrients upon decomposition, but also as agents promoting the physical cohesion of soil [59].

The correlation–regression analysis showed moderate to strong correlations in the top (0–10 cm) layer of soil between the soil structural stability and the content of plant residues on the soil surface in 2014 (r = 0.97; *p* ≤ 0.01) and 2017 (r = 0.99; *p* ≤ 0.01), as well as in the lower soil layer (10–25 cm) in 2014 (r = 0.88; *p* ≤ 0.01).

Our research has unveiled several salient points regarding the impact of tillage practices, especially the role of direct drilling and straw incorporation, on the physical and biological attributes of soil. The intrinsic relationship between plant residues on or within the soil, and its overall quality and fertility, can no longer be understated. The confluence of these factors paints a promising picture for the adoption of conservation tillage methods. However, like all scientific inquiries, these insights come with the caveat that the results might be context-dependent, with other researchers reaffirming the intricacies of soil tillage dynamics [60].

In summary, this research reveals the influence of tillage practices, particularly direct drilling and straw incorporation, on the soil aggregate stability and shear resistance in different soil layers. In addition to plant residues on the soil surface and within the soil, there are essential components that influence the soil quality, fertility, and overall ecosystem health. Their decomposition enriches the soil with nutrients, improving the soil stability. These findings highlight the potential of adopting conservation tillage practices to improve the soil structure and promote sustainable soil management. However, this study also reveals that the impacts of specific tillage practices may vary across different research periods and study conditions. It is important to acknowledge the complexities of soil–tillage interactions and the need for context-specific approaches in agricultural land management.

In conclusion, the results of these studies provide valuable insights for farmers and land managers to make informed decisions regarding good tillage practices that promote soil health, stability, and sustainable land use. Further research and understanding of soil responses to different tillage strategies will contribute to the development of resilient agricultural systems that protect and enhance soil resources for future generations.

## 3. Material and Methods

### 3.1. Site Description

The research was conducted at the Experimental Station of Vytautas Magnus University’s Agriculture Academy (54°52′50″ N latitude and 23°49′41″ E longitude) as a long-term field experiment established in 1999. This study took place in 2014, 2017, and 2023. The soil at the experimental site was classified as Planosol. The long-term experiment was carried out using a split-plot design with four replications, resulting in a total of 24 plots. Initially, each plot had a size of 102 m^2^ (6 × 17 m), and the harvested area measured 30 m^2^ (15 × 2 m).

Table 2 shows the soil characteristics of the experimental plots (16) at a depth of 0–25 cm. The average values for sand, clay, silt, pH_KCl_, soil organic carbon (SOC), available phosphorus (PAL), and available potassium (KAL) are provided [14].

### 3.2. Experimental Design and Agricultural Practices

In an agroecosystem crop rotation, winter oilseed rape (*Brassica napus* L.), winter wheat (*Triticum aestivum* L.), and spring barley (*Hordeum vulgare* L.), which are the most popular crops grown in Lithuania, were chosen. In a two-factor field experiment, the straw (Factor A) in spring barley was removed (R) from one part of the experimental field, and in the other part of the field, the entire straw yield was chopped and spread (S) at harvest. Three different tillage systems (Factor B) were investigated as subplots: (1) conventional deep ploughing (CP) in autumn at a depth of 23–25 cm; (2) cover cropping for green manure with no tillage (GMNT); and (3) no tillage (NT). All of the tillage systems were tested in both halves of the experiment with and without straw. After harvesting, the plots subjected to conventional ploughing were cultivated with disc implements and deep ploughing in autumn. White mustard (*Sinapis alba* L.), a cover crop for green manure on stubble, was sown only in the GMNT plots immediately after the harvest of winter wheat and spring barley.

In 2014 and 2017, the crops were sown with a Väderstad pneumatic no-tillage machine; in autumn 2022, crops were sown with an Agrisem SLY BOSS no-tillage machine. After harvesting the pre-crop (except for winter rape), the straw was removed for one-half of the experiment (R), while for the other half, the straw was chopped and spread (S). All of the tillage systems were tested in both halves of the experiment with and without straw. The design of the experiment and the farming practices are detailed in our previous article [61].

### 3.3. Meteorological Conditions

In 2014, during the vegetation period, the average monthly temperatures were lower than the long-term averages. This indicates that it was relatively cooler during that year, potentially impacting the growth and development of the cultivated plants. Furthermore, the precipitation during this period was unevenly distributed. This means that the amount and timing of precipitation might have varied, which could have had an impact on the water availability and moisture conditions for plant growth.

In 2017, the temperatures (Table 3) at the beginning and the end of the vegetation period were higher than the long-term averages. This suggests that there were periods of relatively higher temperatures, which might have influenced the growth and development of the crops. However, a particularly dry period was observed in June and August, indicating lower-than-average precipitation (Table 4) during these months. This dry period could have hurt the growth of the cultivated plants, as water availability is crucial for plant growth and development.

During the 2023 vegetation period, the average monthly temperatures were very similar to the long-term averages. This implies that the temperatures in that year were within the normal range compared with the long-term climatic patterns. However, it is worth noting that the precipitation over the entire vegetation period decreased less than the long-term average. This suggests that, although there might have been some variations in precipitation, overall, the precipitation in that year was relatively constant compared with the long-term average. This could have contributed to more favorable water conditions for plant growth during the 2022 vegetation period.

Considering these meteorological conditions together with the precipitation data provides a more comprehensive understanding of the environmental factors that might have influenced the growth and productivity of the cultivated plants in that particular year. Furthermore, it was observed that in all of the years studied, the precipitation was lower than the sum of the long-term averages. This suggests a general trend toward lower precipitation during the vegetation period compared with the long-term average conditions. Below-average precipitation can have important consequences for soil moisture availability, water resources, and plant water stress. These conditions can impact the growth, yield potential, and overall health of cultivated plants.

In summary, the climatic parameters during the three-year vegetation period differed and deviated from the long-term average conditions since 1974. This highlights the variability in meteorological conditions and the potential impact on agricultural systems. It is important to consider these climatic variations and their relationship with agrophysical and hydrophysical soil properties. The interaction between meteorological conditions and soil properties is crucial for understanding the overall dynamics of agricultural systems and their response to changing climatic patterns.

### 3.4. Methods and Analysis

Soil sampling and soil aggregate stability analyses were carried out to assess the soil properties and determine the soil aggregate stability. The below procedures were followed.

In the experimental years of 2014, 2017, and 2023, soil samples were collected from the experimental site at two depths in the plough layer: 0–10 cm and 10–25 cm. Ten spots were sampled within each plot to ensure representative soil samples. These individual samples were then combined (250 g per sample) to form a composite sample for each depth, representing the characteristics of the plot.

The collected soil samples were air-dried to remove excess moisture. Subsequently, the soil was sieved using a Retsch sieve shaker (Retsch GmbH, Haan, Germany) to separate it into eight fractions according to diameter: <0.25, 0.25–0.5, 0.5–1.0, 1.0–2.0, 2.0–4.0, 4.0–5.6, 5.6–8.0, and >8.0 mm. Wet sieving apparatus (Eijkelkamp Agrisearch Equipment, Giesbeek, The Netherlands) was used to assess the soil aggregate stability. Specifically, air-dried soil aggregates of 1–2 mm in size were subjected to wet sieving in distilled water. Stable aggregates were retained, while unstable aggregates were destroyed by treating them with a 0.2% (NaPO_3_)_6_ solution. The stable aggregates were then oven-dried at 105 °C for 24 h and weighed. The stability index quantifies the degree of soil aggregate stability and is often expressed as a percentage:

Stability Index (%) = (Weight of Stable Aggregates/Total Initial Weight of Aggregates) × 100

where the Weight of Stable Aggregates is the sum of the weights of the retained stable aggregates after wet sieving and subsequent treatments, and the Total Initial Weight of Aggregates is the sum of the weights of all soil aggregates in the 1–2 mm size range before wet sieving and treatments.

These methods enabled us to determine the soil aggregate stability at depths of 0–10 and 10–25 cm in the plough layer. The analysis provided valuable information on the ability of soil aggregates to resist breakdown under wet conditions, offering insights into structural soil stability.

The procedures described here are consistent with the approach outlined, which provides standardized and reliable measurements of soil aggregate stability [62].

Soil shear strength was assessed using a Geonor 72410 penetrometer (Eijkelkamp Agrisearch Equipment, The Netherlands) at two depths in the plough layer: 0–10 and 10–25 cm. The measurements were conducted after sowing or after the resumption of winter wheat vegetation at 10 different locations within each plot. The penetrometer was used to determine the resistance encountered when applying a controlled force to the soil. These measurements provided insights into the soil’s mechanical strength and its ability to resist shear forces, which are essential for understanding soil stability and its response to different tillage practices and crop management techniques.

The amount of plant residue on the soil surface was determined using a cross-sectional method in two places of each plot. We used a 10 m measuring tape divided into 100 sections every 10 cm. The plots at headland or those with severe symptoms of drying or flooding, severely weed-infested plots, or plots damaged by pests or other adverse factors were excluded from the assessment. The measurements were taken twice in each plot. One end of the measuring tape was fixed, and the other was drawn diagonally across the rows so that it covered several working widths of the tillage machinery. Plant residues that exactly coincided with the marking every 10 cm were counted. Only the residues whose widths were over 2.5 mm were counted. If the 2.5 mm wide marking on the measuring tape did not completely cover the plant residue, the angle was untouched, and the plant residue was not counted.

To determine the amount of plant residue in the soil, soil samples were collected in 10 places in each experimental plot from 0–10 and 10–25 cm plough layer depths after the sowing or resumption of vegetation of winter wheat. Then, the samples were composited. The samples were dried for 24 h at 105 °C. Dry soil samples were transferred into a jar containing water. In the water, the straw was separated from soil clods and was dried again for 24 h in the same temperature, then weighed, and the amounts were calculated for individual layers.

### 3.5. Statistical Analysis

Experimental data were analyzed using a two-factor analysis of variance (ANOVA) based on the methodology in [63] using the SYSTAT statistical software package, version 12 (SPSS Inc., Chicago, IL, USA). The significance of differences among the treatments was determined using the least significant difference (LSD) test. The inter-causality of the tested variables was estimated through the correlation–regression analysis method using STAT ENG software [64]. The probability levels indicating significant differences between specific treatments and the control treatment are denoted as follows: *—when 0.010 < *p* ≤ 0.050 (significant at the 95% probability level); **—when 0.001 < *p* ≤ 0.010 (significant at the 99% probability level); and ***—when *p* ≤ 0.001 (significant at the 99.99% probability level).

## 4. Conclusions

Long-term field experiments conducted over 24 years (1999–2023) provided valuable insights into the changes in the soil physical state resulting from different tillage practices and crop residue incorporation. The findings highlight a significant impact of reduced tillage on the soil shear strength and soil aggregate stability:

1. The long-term application of reduced tillage resulted in a significant increase in the soil shear strength. It was found that the shallower the depth of the tillage, the higher the soil shear strength. The effect of straw retention was lower.
2. The soil aggregate stability was highly dependent on the tillage of the crop residues. The soil aggregate stability increased by up to 2-fold after incorporating the plant residues of white mustard into the soil via no-till practices before sowing, whereas direct drilling (no tillage) increased it by up to 1.9-fold compared with conventional (deep) ploughing.
3. The soil aggregate stability was the lowest when the soil was deeply ploughed every year, while the incorporation of plant residues tended to increase the soil aggregate stability. In the upper (0–10 cm) and lower (10–25 cm) soil layers, direct drilling had a positive effect on the soil aggregate stability.

However, it is important to acknowledge that the results of this study are based on specific experimental conditions, and may not be directly applicable to all soil types, climates, or crops. Further research is needed to validate these findings across different agroecosystems and to establish more comprehensive guidelines for the implementation of reduced tillage practices and crop residue incorporation in diverse agricultural contexts.

## Figures and Tables

**Table 1:** Content of plant residues on the soil surface in 2014 and 2017.

Factors	Content of Plant Residues on the Soil Surface, %	
	Winter Wheat, 2014	Winter Wheat, 2017
R	5.58	6.20
S	11.12 ***	11.34 ***
CP	3.00	2.68
GMNT	13.25 ***	17.38 ***
NT	17.13 ***	24.13 ***

Notes: Significant differences at *** *p* ≤ 0.001; Fisher’s LSD test vs. control. Other explanations are as per Figure 1.

**Table 2:** Soil characteristics of the experimental plot (0–25 cm).

Index	Average Value
Sand %	35.6
Clay %	19.0
Silt %	45.4
pH_KCl_	7.7
Soil organic carbon (SOC) g kg^−1^	16.6
Available PAL mg kg^−1^	116.0
Available KAL mg kg^−1^	111.0

**Table 3:** Average temperature (°C) and the sum of active temperatures (SAT) during the vegetation periods in 2014, 2017, and 2022, measured at Kaunas Meteorological Station.

Year/Month	04	05	06	07	08	SAT
2014	6.1	12.3	15.6	17.6	16.6	1675.6
2017	7.1	11.4	15.4	17.4	20.3	1800.2
2023	9.1	13.0	19.8	17.1	18.1	1918.5
Long-term average, 1974–2023	6.9	13.2	16.1	18.7	17.3	-

SAT, sum of active temperatures (≥10 °C).

**Table 4:** Precipitation (mm) during the vegetation period in 2014, 2017, and 2022, measured at Kaunas Meteorological Station.

Year/Month	04	05	06	07	08	Sum
2014	56.5	63.8	45.9	118.5	67.2	351.9
2017	46.0	43.8	16.4	72.4	6.9	185.5
2022	0.6	29.9	49.4	60.1	68.2	208.2
Long-term average, 1974–2023	41.3	61.7	76.9	96.6	88.9	365.4

## Data Availability

Not applicable.
